# Comparative analysis of mitochondrial genomes of maize CMS-S subtypes provides new insights into male sterility stability

**DOI:** 10.1186/s12870-022-03849-6

**Published:** 2022-10-01

**Authors:** Senlin Xiao, Jingfeng Xing, Tiange Nie, Aiguo Su, Ruyang Zhang, Yanxin Zhao, Wei Song, Jiuran Zhao

**Affiliations:** Beijing Key Laboratory of Maize DNA Fingerprinting and Molecular Breeding, Maize Research Institute, Beijing Academy of Agriculture and Forestry Sciences, Beijing, 100097 China

**Keywords:** Maize, CMS-S, Sterility stability, Mitochondria, Robustness

## Abstract

**Background:**

Cytoplasmic male sterility (CMS) is a trait of economic importance in the production of hybrid seeds. In CMS-S maize, exerted anthers appear frequently in florets of field-grown female populations where only complete male-sterile plants were expected. It has been reported that these reversions are associated with the loss of sterility-conferring regions or other rearrangements in the mitochondrial genome. However, the relationship between mitochondrial function and sterility stability is largely unknown.

**Results:**

In this study, we determined the ratio of plants carrying exerted anthers in the population of two CMS-S subtypes. The subtype with a high ratio of exerted anthers was designated as CMS-Sa, and the other with low ratio was designated as CMS-Sb. Through next-generation sequencing, we assembled and compared mitochondrial genomes of two CMS-S subtypes. Phylogenetic analyses revealed strong similarities between the two mitochondrial genomes. The sterility-associated regions, S plasmids, and terminal inverted repeats (TIRs) were intact in both genomes. The two subtypes maintained high transcript levels of the sterility gene *orf355* in anther tissue. Most of the functional genes/proteins were identical at the nucleotide sequence and amino acid sequence levels in the two subtypes, except for *NADH dehydrogenase subunit 1* (*nad1*). In the mitochondrial genome of CMS-Sb, a 3.3-kilobase sequence containing *nad1*-exon1 was absent from the second copy of the 17-kb repeat region. Consequently, we detected two copies of *nad1*-exon1 in CMS-Sa, but only one copy in CMS-Sb. During pollen development, *nad1* transcription and mitochondrial biogenesis were induced in anthers of CMS-Sa, but not in those of CMS-Sb. We suggest that the impaired mitochondrial function in the anthers of CMS-Sb is associated with its more stable sterility.

**Conclusions:**

Comprehensive analyses revealed diversity in terms of the copy number of the mitochondrial gene *nad1-*exon1 between two subtypes of CMS-S maize. This difference in copy number affected the transcript levels of *nad1* and mitochondrial biogenesis in anther tissue, and affected the reversion rate of CMS-S maize. The results of this study suggest the involvement of mitochondrial robustness in modulation of sterility stability in CMS-S maize.

**Supplementary Information:**

The online version contains supplementary material available at 10.1186/s12870-022-03849-6.

## Background

Cytoplasmic male sterility (CMS) is the maternally inherited inability of plants to produce viable pollen, while plant growth and female fertility are normal [1, 2]. In maize (*Zea mays* L.), there are three major groups of CMS: CMS-C, CMS-T, and CMS-S, which are defined according to the sterilizing factors in mitochondrion and the corresponding main restorers in the nucleus. Maize CMS is an economically valuable trait used for the production of hybrid seeds. The CMS-T system was first used with three-line system technology to produce hybrid maize, which accounted for about 85% of hybrid seeds in the U.S.A. until the 1970 southern corn leaf blight epidemic [3, 4]. Because only one single major restorer (*Rf3*) is required for pollen fertility in the S-system, some breeders transitioned to use CMS-S to produce hybrid maize.

CMS-S maize has a clearly elucidated mitochondria-nucleus interaction system, wherein the sterilizing factor *orf355* initiates microspore degeneration while the single main restorer Rf3 cleaves *orf355* transcripts to restore fertility. CMS-S mitochondria have two linear plasmids known as S1 and S2, which have exactly the same terminal inverted repeats (TIRs) as that in the CMS-associated region. These plasmids actively recombine with the circular genome to linearize the mitochondrial DNA (mtDNA) [5–8]. The gene encoding the sterilizing factor *orf355* is located near the end of the linearized mtDNA and is expressed at the bi-cellular stage of microspore development, leading to the gametophytic sterility of maize [9, 10]. In the presence of *Rf3*, the 1.6-kb transcripts containing *orf355* are cleaved via posttranscriptional modification, leading to reduced *orf355* transcript levels and the restoration of fertility [11].

However, CMS-S maize is a relatively unstable system where revertants frequently arise as a result of genetic mutation [12]. In the absence of *Rf3*, sterility reversions are caused by less effective genetic restorer-of-fertility genes, or by the mtDNA changes that usually arise in de novo [8, 9, 13, 14]. Although the reversions usually take place in a single floret or a small sector of the tassel, they can potentially reduce the purity of hybrid seeds. This can hinder the use of CMS-S for hybrid seed production. Unstable sterility usually occurs in the absence of the main restorer Rf3. For example, nuclear-encoded restorer Rf9 does not cause the cleavage of the 1.6-kb transcripts but decreases the abundance of the linearized transcripts of the mtDNA template [8]. In addition, the loss of the free S1 and S2 plasmids from the mitochondria blocks the rearrangement with the sterility-associated region, leading to the restoration of fertility in CMS-S maize [9]. In some cases, mtDNA rearrangement disrupts the sterility-associated region so that the sterilizing factor is either lost or not expressed. For example, some revertants have a 7.3-kb inversion in the mtDNA that separates the TIR sequences from the CMS-associated region [13]. As a result, *orf355* is no longer transcribed from mtDNA linear ends but is co-transcribed with *cox2*. Although *orf355* transcripts can still be detected in these plants, they are not highly expressed and no longer confer sterility [13]. The *restorer-of-fertility lethal 1* (*rfl1*) mutant disrupts mitochondrial gene expression and the accumulation of the α-subunit of ATP synthase, suggesting that the functional plasticity of mitochondria is linked with the male sterility stability of CMS-S [14].

The maize mitochondrial genome is about 500 kb in size, and contains a suite of relatively conserved protein-encoding genes within different cytotypes [15]. The relative placement of the genes and the intergenic spacer regions within the mitochondrial genome vary extensively among different maize subgroups [16]. A major reason for the highly variable structural organization of the maize mtDNA is the abundance of recombination-active repeated sequences [17]. The shuffling of mtDNA sequences by recombination plays a role in evolution, and changes gene organization and creates gene chimeras [16]. All three types of CMS in maize, as well as CMS systems in other species, are caused by chimeric open reading frames (ORFs) arising from mtDNA rearrangements. Mitochondrial rearrangements associated with the loss of portions of essential genes cause poor growth or lethality in maize [18, 19]. In contrast, rearrangements affecting only non-coding regions of mtDNA are usually neutral, but sometimes cause different phenotypes, such as fertility reversion in CMS-S maize [20–22]. Maize mitochondrial genomes have a special type of DNA sequence acquired from the plastid, which accounts for 4.4% of CMS-S mitochondrial genome [23]. The plastid-derived DNA sequences vary widely in their content and location among the five maize mitochondrial genomes [15]. However, the relationship between the plastid-derived DNA sequences and recombination is unclear.

The mitochondrion is a semi-autonomous organelle, which is capable of responding to fluctuating energy demands and environmental stimuli [24]. Mitochondrion is capable of maintaining a constant ATP to ADP ratio over a wide range of conditions. This key property is referred to as mitochondrial robustness, and can explain why some even severe mutations can be tolerated within the oxidative phosphorylation system and be compatible with life. Studies on animals suggest that the tissue-specific control coefficients of different respiratory chain complexes counteract the influence of mitochondria, and probably vice versa [25]. Plant CMS might result from the inability of mitochondria to meet the increased energy demands of microspore development [2]. However, the relationship between mitochondrial robustness and sterility determination is largely unknown. Here we describe features of the mitochondrial genome of a CMS-S subtype that has retained an intact *orf355*/*orf77* region but has lost the second copy of *nad1-*exon1. This change was found to reduce mitochondrial biogenesis and gene transcription in anther tissue, leading to stable male sterility in CMS-S maize.

## Materials and methods

### Plant materials and growth conditions

The subtype CMS-Sa was originated from male sterile line S-Mo17^*rf3rf3*^ [26] and were donated by professor Yonglian Zheng in Huazhong Agricultural University (Wuhan, China). The subtype CMS-Sb were originated from male fertile line S-SD13^*Rf3Rf3*^ [10] and were donated by professor Huabang Chen in Institute of Genetics and Developmental Biology, Chinese Academy of Sciences (Beijing, China). The sterility of both lines can be counteracted by the Rf3 restorer. Jing72464 is the female parent of an elite hybrid line in China, which was developed by the Maize Research Institute, Beijing Academy of Agricultural and Forestry Science (Beijing, China). Both subtypes were introduced into Jing72464 to develop the sterile inbred line. By crossing two cytoplasm donors with the fertile Jing72464 (NB), the sterile F_1_ was obtained, which was consecutively backcrossed with Jing72464 for more than 10 generations. Molecular markers were used to select the Jing72464 nuclear background in each backcross generation, so that the sterile families in the two CMS-S subtypes can be considered as near isogenic lines. The plant materials were cultivated at the Hainan maize propagation base in Yacheng, Hainan (HN-YC, 18.3°N, 109.5°E) and a farm near Beijing (40.1° N, 116.4° E).

### Pollen viability test

A fertility survey was conducted at the Beijing farm (40.1° N, 116.4° E) in the summer of 2021. During flowering, every plant was observed every day to detect exerted anthers. Plants with even one exerted anther in any sector of the tassels were counted. Pollen viability was checked using the iodine-potassium iodide (I_2_-KI) staining method. The pre-exerted anthers were cut with tweezers in 50 μL I_2_-KI solution on a glass slide, and then large debris were carefully removed. The stained pollen grains without a cover slip were directly observed under a light microscope at 20× magnification as described previously [27]. At least 100 pollen grains per visual field were counted randomly under a light microscope. Successful seed set indicated that pollen was fertile. Pollen grains were carefully collected from the exerted anthers and crossed onto ears of CMS-Sb Jing72464 inbred plants to confirm pollen fertility. In some cases, anthers were cut with tweezers and the pollen was applied directly to filaments. Progeny families from the above tests were cultivated and their fertility was checked to determine whether the restoration of pollen fertility could be transmitted via the pollen.

### Library construction and sequencing

Mitochondrial DNA was extracted as described previously [28]. Ten-day-old etiolated seedlings were homogenized in homogenization buffer (0.4 M mannitol, 10 mM TES pH 7.5, 5 mM EGTA, 0.05% cysteine, 0.2% BSA) in a blender, then the mixture was filtered through four layers of cheesecloth and one layer of Miracloth. Crude mitochondria were obtained by differential centrifugation (1000 g, 5 min; 2000 g, 10 min; 10,000 g, 20 min). The crude mitochondrial pellet was suspended in 2 mL homogenization buffer, then purified using sucrose step gradient centrifugation. The sucrose step gradient was established using sterile sucrose solutions (60, 47, 35, and 20% w/v sucrose in 10 mM tricine, 1 mM EGTA, pH 7.5) in ultraclear tubes. The resuspended mitochondria were carefully layered onto the sucrose gradient and centrifuged at 30,000 rpm (about 111,000 *g*) in a SW41 rotor for 60 min at 4 °C. The mitochondria were condensed at the interface between the 35 and 47% sucrose layers. The collected mitochondria were diluted with three volumes of homogenization buffer. Purified mitochondria were obtained by centrifuging the mixture at 10,000 g for 30 min at 4 °C. Then, DNA was extracted from the purified mitochondria using a DNeasy plant mini kit (QIAGEN, Hilden, Germany).

The libraries were constructed with the TruSeq Nano DNA LT Sample Preparation Kit (Illumina, San Diego, CA, USA). Briefly, the mitochondrial DNA was sheared into fragments with lengths of approximately 350 bp using a S220 Focused ultrasonicator (Covaris, San Diego, CA, USA). Adapters were ligated onto the 3′ end of the sheared fragments. After PCR amplification and purification, the final libraries were sequenced on the Illumina sequencing HiSeq X Ten platform (Illumina) and 150-bp paired-end reads were generated. The sequence data were de novo assembled using ABySS [29] with different k-mer values (k = 31 to 127, increasing step-wise by 2 each time) to choose the best k-mer value for assembly. To verify the quality and accuracy of our assemblies, the original reads were mapped to the corresponding mitogenomes using BWA [30]. Pilon was used to polish the final assembly. Mitochondrial protein-coding genes were predicted using GeSeq [31], and then the start/stop codons and the exon-intron boundaries of genes were revised manually. The GC content was determined with a shell script and the circular physical map of all mitogenomes was visualized using OGDRAW [32]. The Synteny and single nucleotide polymorphism (SNP) analyses of the different mitogenomes were conducted using MUMmer [33].

### Phylogenetic analysis

To understand the phylogenetic position of two CMS-S subtypes, the mitochondrial genomes of CMS-Sa, CMS-Sb, and DQ490951 were used to build an evolutionary tree, mitochondrial genome of Sorghum (DQ984518) was used as the outgroup. Phylogenetic analysis was based on nucleotide sequences of the 4 mitochondrial genomes. These nucleotides were aligned using MAFFT [34] and gaps were removed from the alignments using the trimAL [35]. A maximum likelihood (ML) tree was constructed using IQ-TREE [36] with a Best-fit model from ModelFinder [37] and bootstrap consensus was inferred from 1000 replications. The tree was finally drawn using R script.

### Analysis of mitochondrial plastid DNAs and the corresponding plastid homolog

A total of 11 pairs mitochondrial plastid DNA and plastid homologs were selected and compared based on previous study [15]. Fragments covering the target sequences were amplified from genomic DNA isolated from ten-days-old seedlings using suites of specific primers. Then the fragments were sequenced by Sanger sequencing. The primers for the amplification of these fragment are listed in Additional file 1: Table S1.

### Quantification of mitochondrial DNA

Mitochondrial DNA in maize seedlings was quantified as described previously [38] with slight modifications. Total DNA was extracted from a pool of five ten-day-old maize seedlings leaves, or from the anthers collected from pre-emerged tassels. A total of 40 ng DNA was analyzed by real-time PCR using a Light Cycler 480 (Roche, Basel, Switzerland). The reaction mixture (final volume, 20 μL) contained SYBR green master mix (Roche) and 1.25 mM forward and reverse primers. The proportion of mitochondrial DNA out of total DNA was determined using the primer pairs C33–C34 and C35–C36, then normalized to the internal standard nucleus-encoded single-copy fragment C39–C40 as described previously [38]. Each data point represents the average of at least three technical replicates. Primer sequences are shown in Additional file 1: Table S1.

### RNA extraction and gene transcript level analysis

Whole seedlings with the first expanded true leaf were sampled from each subtype. Anther tissue was carefully collected from the florets of pre-emerged tassels. Total RNA was isolated using a Plant RNA Purification Kit (K0801, Thermo Fisher Scientific, Waltham, MA, USA). Full-length cDNA was generated from 2 μg total RNA per sample using random primers (cDNA synthesis kit, K1622, Thermo Fisher Scientific). The resulting cDNA was diluted 1:10 for quantitative RT-PCR (qRT-PCR) analyses, which were conducted using the TB Green® Premix Ex Taq™ II Real-Time PCR System (RR820A, Takara, Otsu, Japan). Each 20 μL PCR mixture consisted of 10 μL premix, 3 μL template cDNA, and 0.4 μM each PCR primer. The thermal cycling conditions were as follows: 95 °C for 2 min, followed by 40 cycles at 95 °C for 20 s, 56 °C for 1 min, and 72 °C for 30 s. Samples were run on the LightCycler 480 Real-Time PCR System (Roche). Melting curve data were used in every experiment to verify amplification of the appropriate PCR product. The mitochondrion-encoded gene *rrn18* was used as the internal control to monitor sample uniformity of initial RNA input and reverse transcription efficiency. The 2^−ΔΔCt^ method was used to calculate the relative transcript levels of mitochondrial genes. The primer sequences are listed in Additional file 1: Table S1.

### Circular RT-PCR analyses

Circular RT-PCR (cRT-PCR) analyses were conducted to detect the primary and processed transcripts of *nad1* and *nad2* from the 17-kb repeat region. The extracted RNAs were treated with RNA 5′ polyphosphatase (#M0356, NEB, Boston, MA, USA) before self-ligation. The circular cDNA was reverse-transcribed using gene-specific primers. Two-round PCR was conducted to amplify the sequences flanking the 5′ and 3′ extremities. The products of second-round PCR were separated by agarose gel electrophoresis, and the bands of expected size were sequenced by cloning into a vector. These procedures were conducted as described elsewhere [39] and according to the manufacturer’s instructions. The primers used for reverse transcription and cRT-PCR amplifications are listed in Additional file 1: Table S1.

## Results

### Different sterility stability in two subtypes of CMS-S maize

**Fig. 1:**
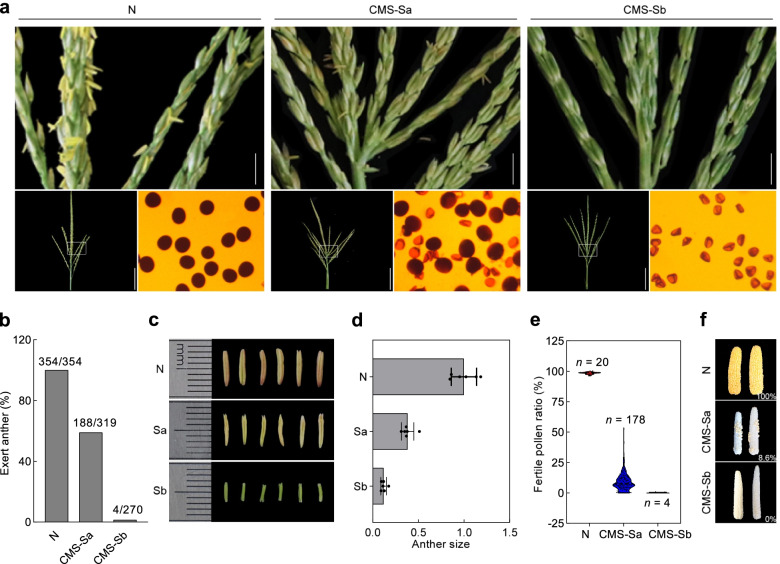
Different sterility stability in two subtypes of CMS-S maize. **a** Pictures of tassel sectors on cytoplasm N, CMS-Sa, and CMS-Sb. Scale bar, 1 cm. The lower left panel show full image of each tassel. Scale bar, 10 cm. The lower right panel show representative pollen grains that stained with I-Ki solution in each subtypes. **b** Spontaneous reversion rate of CMS-S subtype Sa and Sb, plant with exerted anther in floret was considered as spontaneous reverted individuals. **c** Image of anthers in CMS-Sa and CMS-Sb. Scale bar, 1 cm. **d** Anther size calculated using *Image J*. Data are mean ± s.e.m. (*n* = 6). **e** Fertile pollen ratios, calculated as the ratio of darkly stained pollens versus the lightly stained pollens in each plantlets. **f** Tests of pollen viability, as illustrated by seed setting rate in representative ears pollinated using pollen from each subtypes

In the hybrid breeding program, two subtypes of CMS-S (designated as CMS-Sa and CMS-Sb) were used as the cytoplasm donors to generate sterile female parents of elite maize hybrids (see methods). Of the 319 plants derived from subtype CMS-Sa, 188 had visible anthers exerted from the florets. These anthers were smaller and usually emerged from the basal region of the tassel sectors after natural pollination (Fig. 1 a-d). The male sterile stability was significantly higher in CMS-Sb than in CMS-Sa (only 4 of the 270 progenies produced visible anthers at the same stage). The anthers in subtype Sb were severely shrunken, implying that there was no viable pollen in the locules (Fig. 1a-d). Pollen development was checked via the I_2_-KI staining method. Among the 178 analyzed anthers from CMS-Sa, most (170/178) produced a small amount of stainable pollen (Fig. 1e). In contrast, none of the four analyzed anthers from CMS-Sb produced stainable pollen. To test the pollen viability of CMS-Sa, revertants were crossed as pollen parents onto ears of pollen-sterile testers. Twenty-three families were produced with successful seed set, indicating that the pollen was indeed viable (Fig. 1f). However, the seed setting rate was very low, which less than 10% in the representative ears. Notably, natural pollination of these plants had already finished by the time the anthers were exerted from the florets, implying that fertility reversion was only partial and the leaked pollen grains were able to meet the unfertilized silks to complete fertilization only rarely. We speculated that, because the experimental materials were near isogenic lines, variation in fertility reversion was likely due to non-nuclear differences. To test this idea, 15 of the above 23 families with good seed set were grown and their pollen fertility was evaluated. As expected, none of the plants were fertile or partially fertile, indicating that no nuclear restorer allele had been transmitted from the pollen to the next generation.

### Mitochondrial genomes of two CMS-S subtypes

**Fig. 2:**
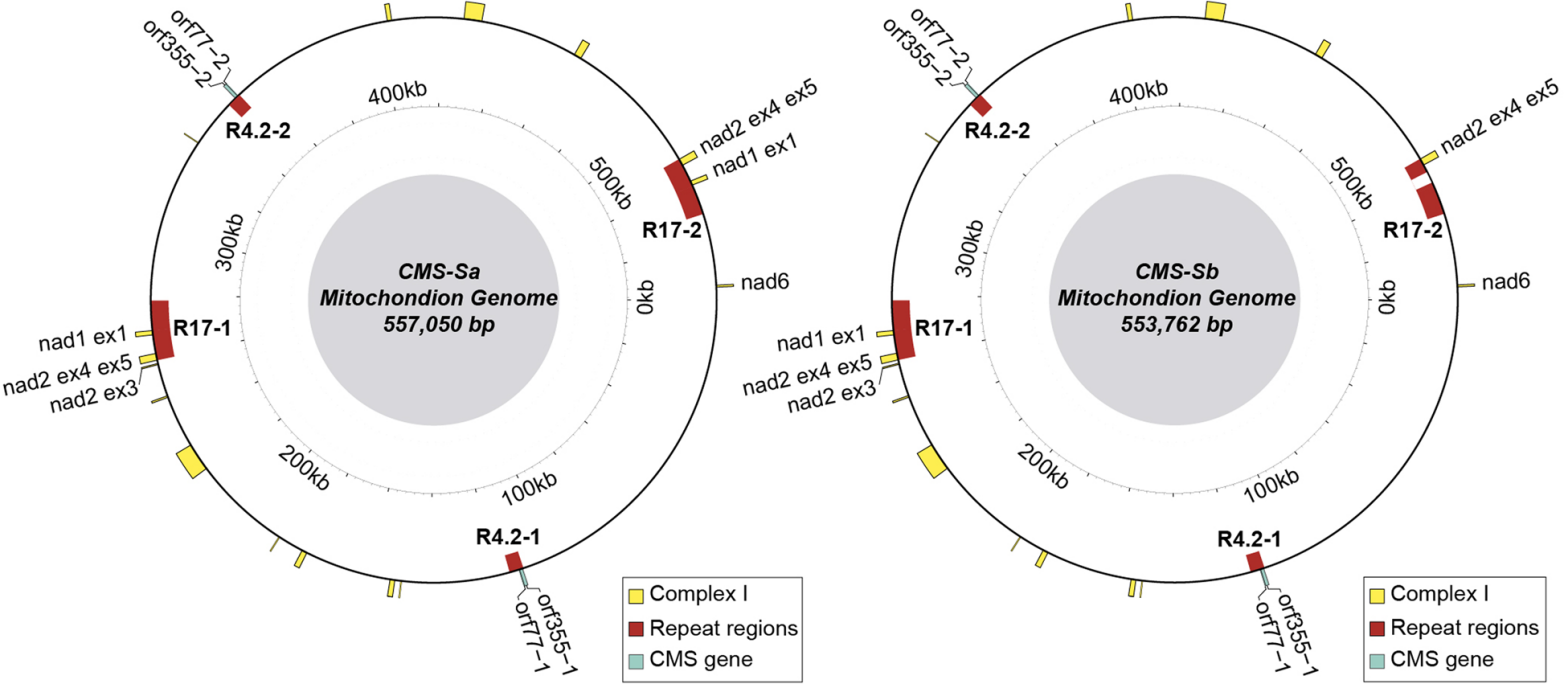
Graphical genome maps display major difference between CMS-Sa and CMS-Sb. Both genomes have identical sequence of 4.2-kb repeat regions that contains the sterilizing gene orf355. The second copy of 17-kb repeat of CMS-Sb lost 3.3-kb sequence, leading to the elimination of one copy of first exon of nad1. Yellow rectangles represent coding region of nad1. Repeat regions sequences were labeled in brown color

Because mitochondrial genome structure, gene organization, and linearization all affect sterility, the mitochondrial genomes of the two CMS-S subtypes were sequenced from highly purified mitochondrial DNA. Previous studies have demonstrated that the majority of the CMS-S genome is present in the linear form [6]. However, in this paper, the assemblies of the subtypes’ genomes are depicted in circular form for better illustration. The two newly sequenced CMS-S subtype genomes showed high similarity with each other and with the reference genome [15] (Additional file 2: Fig. S1), but exhibited variable sizes with 557,050-bp in CMS-Sa and 553,762-bp in CMS-Sb (Fig. 2). Variations in large repeats (> 0.5-kb) accounted for the majority of the size differences between the mitochondrial genomes of subtype CMS-Sa and CMS-Sb. There are 22 predicted large repeats in the mitochondrial genomes of CMS-S maize [15]. The two newly sequenced mitochondrial genomes shared the same size and copy number of all repeats except for a 17-kb repeat region designated as R17. The first copy of R17 was identical among CMS-Sa, CMS-Sb, and the reference genome, whereas the second copy of R17 had a deletion of a 3.3-kb sequence from its internal region in the mitochondrial genome of CMS-Sb (Additional file 3: Fig. S2 and Additional file 4: Fig. S3). Most of the functional genes/proteins were identical at both the nucleotide and amino acid sequence levels in the two subtypes. However, *apocytochrome b* (*cob*) and *NADH dehydrogenase subunit 1* (*nad1*) differed between the genomes of the two subtypes. The *cob* gene of CMS-Sa had a T to G substitution at position + 312, resulting in a predicted amino acid change from phenylalanine to leucine. There were two copies of *nad1*-exon1 in CMS-Sa, but only one copy of *nad1-*exon1 in CMS-Sb because of the deletion of the 3.3-kb sequence.

### Nucleotide substitutions mainly occur in the plastid-derived sequence in newly sequenced CMS-S subtypes

Alignments with the reference genome revealed 117 and 104 nucleotide substitutions in CMS-Sa and CMS-Sb, respectively. The nucleotide substitution frequency in CMS-Sa (2.1 substitutions/10,000 bp) was higher than that in CMS-Sb (1.88 substitutions/10,000 bp) (Additional file 5: Table S2). It is thought that there is a lower frequency of indels than substitutions in plant mitochondrial genomes [15]. Consistent with this, the CMS-Sa and CMS-Sb mitochondrial genomes had a low ratio of indels to substitutions: 0.17 and 0.15 indels/substitution relative to the reference genome, respectively. More than half of the indels were five nucleotides in length. Length variations in simple sequence repeats (SSRs) accounted for 41% of the total indels (Additional file 6: Table S3 and Additional file 7: Table S4).

**Fig. 3:**
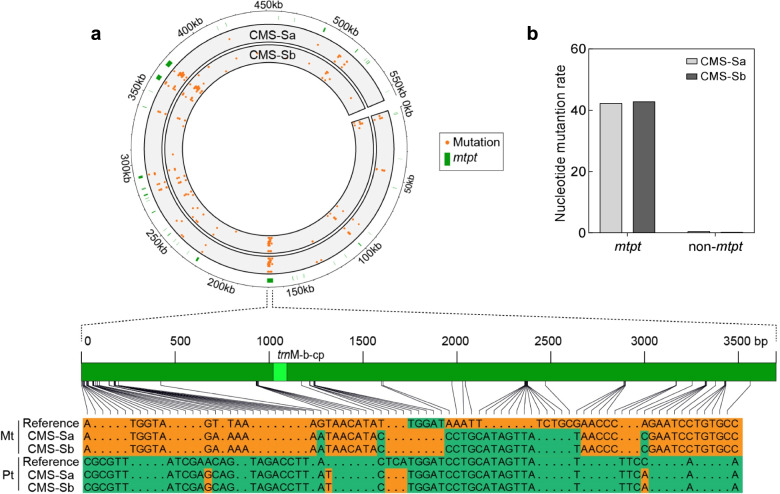
Nucleotide substitutions mainly occurred in the plastid-derived sequence in new sequenced CMS-S subtypes. **a** Mutation bias in plastid-derived DNA sequences (*mtpt*) of CMS-S mitochondrial genome. Orange dots in upper panel represent nucleotides mutation in each subtypes that compared with referee. *Mtpt* sequences are labeled in green color. Lower panel shows individual nucleotide mutations in the representative 3658-bp *mtpt* sequence and chloroplast homolog among NB, CMS-Sa, and CMS-Sb. Mt, mitochondrial; Pt, plastid. **b** Mutation rate in *mtpt* and non-*mtpt* sequence of CMS-Sa and CMS-Sb, calculated as the number of substitution or indel per 10,000 nucleotide

Compared with the reference genome, CMS-Sa and CMS-Sb shared 92 identical nucleotide substitutions. These substitution sites were not evenly distributed, but clustered in several hot spot regions (Fig. 3a). Notably, most of these hot spots were in plastid-derived sequences (mitochondrial plastid DNAs: *mtpt*). For example, 22 of the substitutions were within the 3726-bp *mtpt* sequence that was present in the mitochondrial genome of both subtypes. However, the corresponding 3681-bp homolog sequences in the plastid were almost identical among CMS-Sa, CMS-Sb, and the reference plastid genome (NC_001666.2) (Fig. 3a). To eliminate the possible interference of plastid genomes during sequencing and assembly, we amplified these *mtpt* and their corresponding plastid sequences separately from the mitochondrial genome and the plastid genome using suites of specific primers. The results indicated that the analyzed *mtpt* sequences in CMS-Sa and CMS-Sb were different from their ancestral homolog sequences in the plastid. Thus, nucleotide substitutions have occurred in the mitochondrial genomes of CMS-Sa and CMS-Sb, especially in plastid-derived sequences. The substitution rates in *mtpt* sequence were about 42.3 and 42.8 substitutions/10,000-bp in CMS-Sa and CMS-Sb, respectively, significantly higher than the rates in non-*mtpt* sequences (Fig. 3b). Most of the clustered substitution sites surrounded the functional plastid-originated tRNA coordinates (Additional file 5: Table S2).

### Transcript levels of *orf355* in the two CMS-S subtypes

**Fig. 4:**
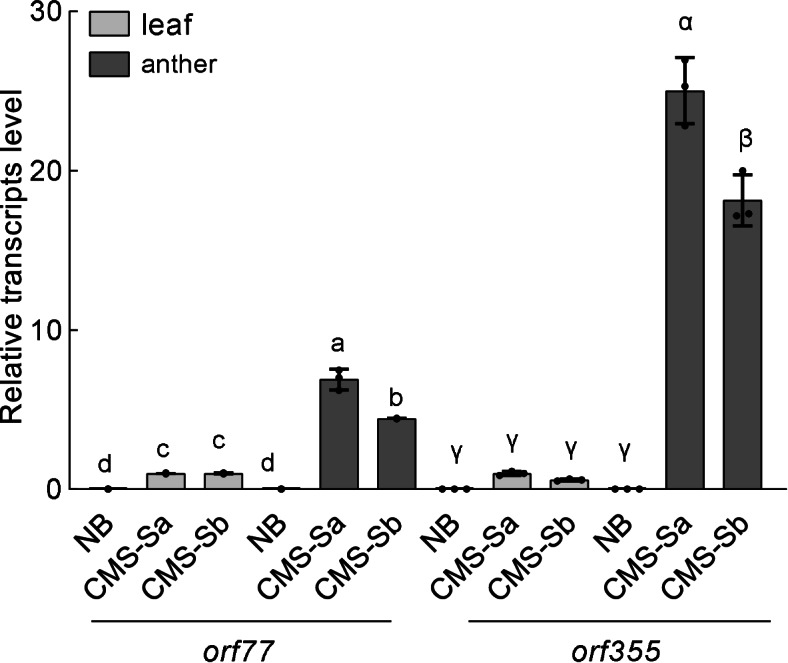
Transcript level of sterilizing gene in CMS-Sa and CMS-Sb. Transcript level of orf355 and *orf77* in leaf and anther in NB, CMS-Sa, and CMS-Sb. Data are mean ± s.e.m. (*n* = 3). Different letters denote significant differences determined by Tukey’s tests, *P* < 0.05

Next, we focused on differences in the sterility-associated region between the two CMS-S subtypes. In the CMS-S mitochondrial genome, there are two copies of a 4.2-kb repeat (R4.2) containing *orf355*-*orf77* sequences. The second copy of R4.2 located downstream of *cox2* recombines with S plasmid to yield linearized CMS-S genomes, from which the sterilizing 1.6-kb RNA is transcribed [8]. Previous studies demonstrated that expression of *orf355-orf77* is associated with the male sterility of maize CMS-S. Sequencing of the coding region revealed that the DNA sequence of R4.2 was identical in two subtypes (Fig. 2). Most spontaneous reversions in CMS-S maize are associated with the integrity of the free S plasmid and TIR sequence of main mitochondrial genome. However, the newly sequenced subtypes CMS-Sa and CMS-Sb shared identical intact S1, S2 and TIR sequences with the reference sequences [40, 41]. Next, we checked the transcript levels of *orf355* and *orf77* in the leaves and anthers of the two accessions, normalized against the mitochondrial rRNA gene, *rrn18*. Quantitative RT-PCR analyses demonstrated that the *orf355* transcript levels were significantly higher in the anthers than in the leaves in both subtypes. The transcript abundance of *orf355* was even slightly lower in CMS-Sb than in CMS-Sa (Fig. 4). Thus, other factors beside the sterilizing factor may also influence the stability of sterility in CMS-S maize.

### Similar post-transcriptional processes in the R17 region in CMS-Sa and CMS-Sb

**Fig. 5:**
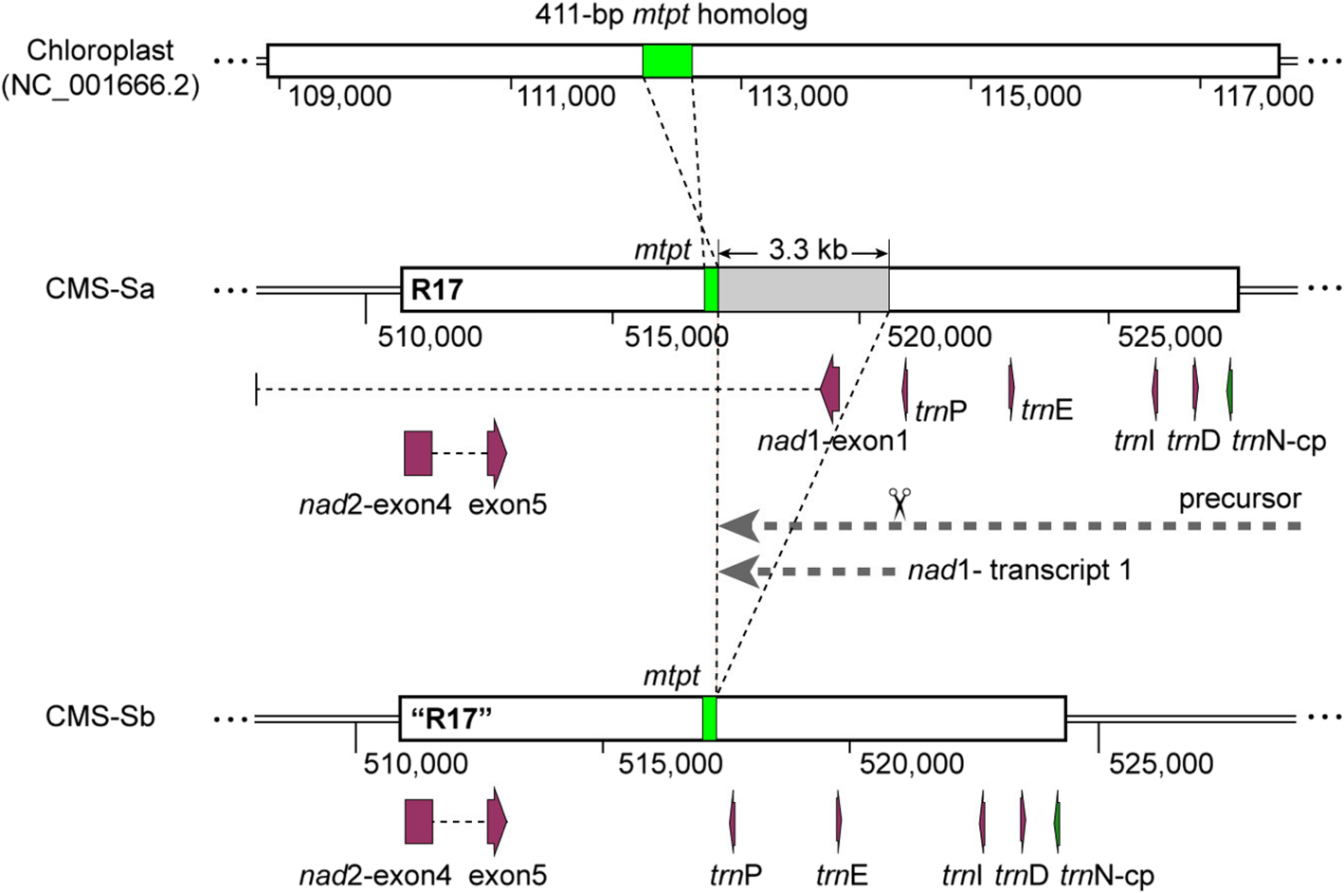
Schematic map of the second copy of 17-kb repeat in CMS-Sa and CMS-Sb. Coding genes are indicated by boxes of purple color. The *mtpt* sequence and its homolog in chloroplast genome are labeled in green color. The eliminated 3.3-kb sequence in CMS-Sb is labeled in gray color. The putative *nad*1 transcripts were cleaved at the sites adjacent to *trn*P to generate the 5′ end of precursor. 3′ end of nad1 precursor were tested at the sites adjacent to *mtpt* sequence

**Fig. 6:**
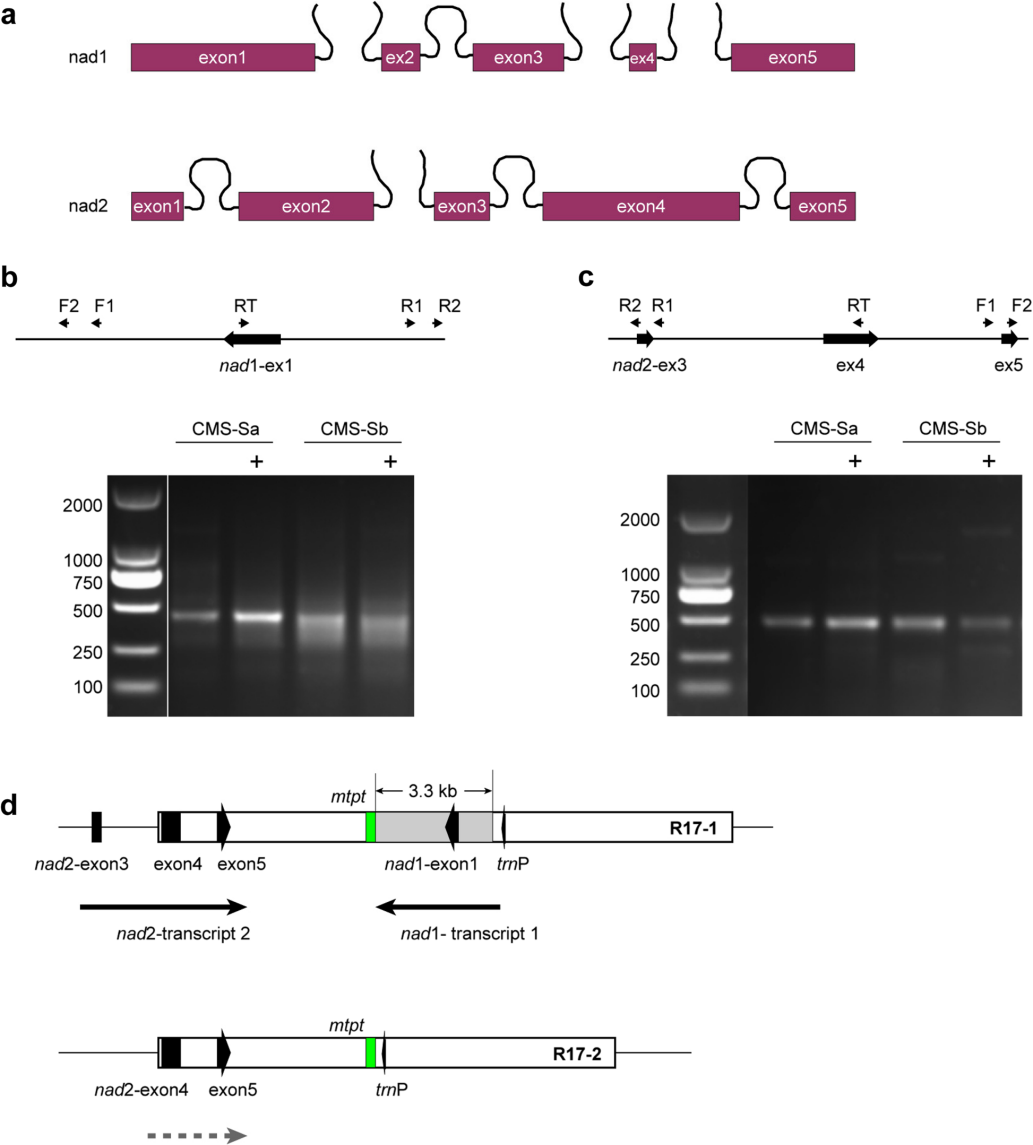
Similar post-transcriptional process in R17 region was observed in CMS-Sa and CMS-Sb. **a** Diagrammatic representation of *nad1* and *nad2* RNA transcripts that generated from post-transcriptionally processing of the precursor. **b** and **c** The cropped gels of cRT-PCR products of *nad1*-transcripts 1 (**b**) and *nad2*-transcripts 2 (**c**). “+” indicated RNA samples treated with RNA 5′-polyphosphatase. **d** Schematic map of two copies of R17 in CMS-Sb. Circular RT-PCR (cRT-PCR) in CMS-S revealed that *nad1*-exon1 were processed downstream of *trn*P to generate the 5′ terminus and upstream of *mtpt* sequence to generate the 3′ terminus. No obvious difference was observed in the fragment length and processing site of cRT-PCR products between subtype CMS-Sa and CMS-Sb, which due to the presence of intact first copy of R17 is both genomes. cRT-PCR analysis revealed similar precursor of *nad2*-exon3, exon4, and exon5 that flanking border of the first copy of R17. Consistent with previous work of Zhang et al, no specific precursor that flanking nad2-exon4 and exon5 in the second copy of R17 were identified, which probably due to low abundance of this precursor

The 3.3-kb deletion in the second copy of R17 accounted for the major differences between the two CMS-S subtypes. R17 contains several functional genes, including five tRNA genes, the first exon of *nad1*, and the fourth and fifth exons of *nad2*. Notably, a 411-bp *mtpt* sequence was located within the intergenic region between *nad2*-exon5 and *nad*1-exon1. The site of the deletion in CMS-Sb was between the *mtpt* sequence and *trnP*, leading to the elimination of *nad*1-exon1 (Fig. 5). In the maize mitochondrial genome, *nad*1 and *nad*2 were interrupted by group II introns (Fig. 6a). Mature functional RNA is generated through posttranscriptional modification of the corresponding precursors [23]. A previous study using NB mitochondrial maize illustrated that *nad*1-exon1 is co-transcribed with upstream tRNAs to generate polycistronic precursor RNAs [39]. Following endonucleolytic cleavages at specific sites adjacent to tRNAs, mature RNA of *nad*1-exon1 is released. R17 is present in all cytotypes of maize except for CMS-T [15], which implies that CMS-S probably has identical transcripts to those of NB maize. We performed cRT-PCR to explore the post-transcriptional processing of genes in R17. The results revealed that *nad1*-exon1 was processed downstream of *trnP* to generate the 5′ terminus and upstream of the *mtpt* sequence to generate the 3′ terminus (Fig. 6b). Because the first copy of R17 was intact in both subtypes, there was no difference in the fragment length and processing site of cRT-PCR products between them (Fig. 6b and Additional file 8: Fig. S4a). Similarly, cRT-PCR analysis only amplified transcripts flanking *nad2*-exon3, exon4, and exon5 adjacent to the first copy of R17. No *nad2* transcripts specific to the second copy of R17 were identified (Fig. 6c-d and Additional file 8: Fig. S4b). Thus, according to our results, there was no obvious differences in post-transcriptional processing between CMS-Sa and CMS-Sb.

### Sterility-stable subtype CMS-Sb displayed reduced mitochondrial function in anthers as compared with CMS-Sa

**Fig. 7:**
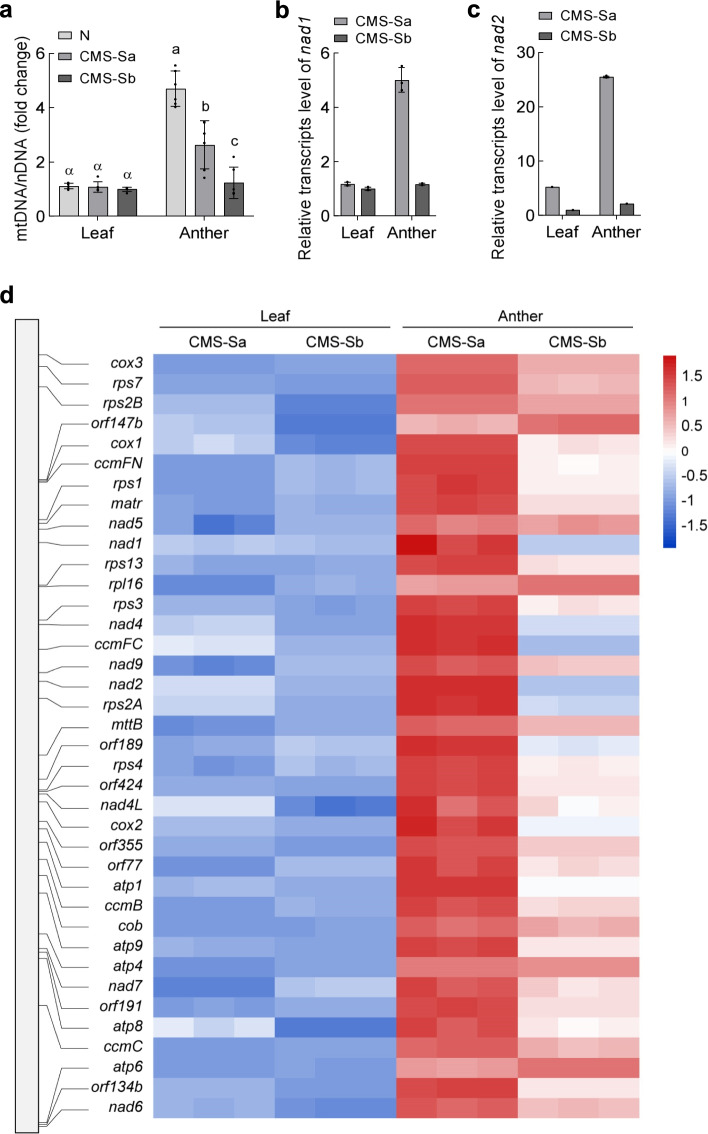
The sterility-stable subtype CMS-Sb displayed reduced mitochondrial function in anther as compared CMS-Sa. **a** Mitochondria biogenesis in leaf and anther of fertile, CMS-Sa, and CMS-Sb, as illustrated by relative abundance of mitochondrial DNA versus nuclear DNA. Mitochondrial DNA levels were measured by two independent primer pairs of mitochondria, then normalized to the internal standard nucleus-encoded single-copy fragment. Data are mean ± s.e.m. (*n* = 6). **b** and **c** Transcripts abundances of representative mitochondria encoding genes *nad1* (**B**) and *nad2* (**C**) of Complex I. Abundances shown are relative to the level of CMS-sb (set to 1). Data are mean ± s.e.m. (*n* = 3). **d** Relative transcripts levels of mitochondria encoding genes in leaf and anther tissues. Transcripts levels were normalized to the minimum means among four groups (set to 1). The relative positions of genes as indicated by horizontal lines in the mitochondrial schematic map of CMS-S

Mitochondrial function increases in anthers to meet the large energy demands of developing pollen. We determined the ratio of the mitochondrial genome to the nuclear genome to estimate the average mitochondrial copy number per cell in different tissues. The results of qRT-PCR analyses suggested that, on average, there were more mitochondrial genome copies per cell in the anther tissues than in seedlings (Fig. 7a). The mitochondrial copy number in anthers was lower in CMS-Sb than in CMS-Sa, implying that mitochondrial biogenesis was impaired in CMS-Sb (Fig. 7a). Transcript levels of mitochondrial genes were also analyzed. The transcript levels of most mitochondrial genes were significantly increased in anthers of CMS-Sa, but not in anthers of CMS-Sb (Fig. 7d). For example, the transcript levels of *nad1* and *nad2* were more than four times higher in anthers than in seedlings of CMS-Sa, but not significantly different between anthers and seedlings in CMS-Sb (Fig. 7b, c). These results suggest that lower mitochondrial function in CMS-Sb maize during pollen development enhances the stability of its sterility.

## Discussion

The mitochondrion is a dynamic organelle that modulates their function and biogenesis in response to fluctuating energy demands triggered by developmental signals and environmental stimuli [24]. One key property of mitochondrion is its robustness, that is the ability to maintain the respiration rate and ATP synthesis over a wide regime, even in face of some severe mutations in the mitochondrial genome. Robustness of mitochondrion has important implications for understanding why some mutations in mitochondrial genomes are lethal and some only manifest in specific tissues [24]. Working models to explain such phenomena have been proposed on the basis of results from studies on animals [25]. One explanation is that the consequences of defects in oxidative phosphorylation (OXPHOS) complexes depend on severity of the defect and the biological threshold of the tissue. Thus, in a given tissue with high biochemical threshold, even a small quantity of normal mitochondria are sufficient to maintain a normal level of oxidative phosphorylation. Conversely, in another tissue with a low biochemical threshold, a very small decrease in OXPHOS function can induce mitochondrial collapse [25]. This hypothesis can explain the unstable sterility in CMS-S maize.

The number of mitochondrial copy number per diploid cell is large and highly variable during plant development, whereas the copy number in the nucleus remains essentially constant [42]. According to early proposal, high copy number of mtDNA reflects an increased demand for organellar ribosomes that can only be satisfied by increased rRNA gene number that results from genome amplification [43]. This probably reflect the capacity of mitochondrion to regulate gene expression in response to changed physiological condition. Studies on animals suggest that the increased mtDNA copy number is associated with ROS-generated oxidative stress [44]. Previous study detailed the ROS content change during pollen development [10]. Plant microsporogenesis is an energy-demanding process, but ATP required for pollen development is supplied mainly by mitochondria, which are non-photosynthetic [45]. Thus, large amount ROS were generated as inevitable byproduct of oxidative phosphorylation. Previous studies have reported that induction of mitochondrial biogenesis can result in a 20- to 40-fold increase in some anther cells in maize, and that this is accompanied by increased expression levels of several mitochondrial genes in microspores [45, 46]. We detected increased mitochondrial copy number and genes expression in CMS-Sa, although lower than those in the same tissues of NB. Therefore, anther tissue of CMS-Sa maintains considerable mitochondrial robustness. However, we cannot exclude the possibility that the increased mtDNA copy number is caused by *orf355*-mediated mitochondrial degradation, which also associated with the increase of mtDNA content. Further studies are needed to dissect this mechanism.

**Fig. 8:**
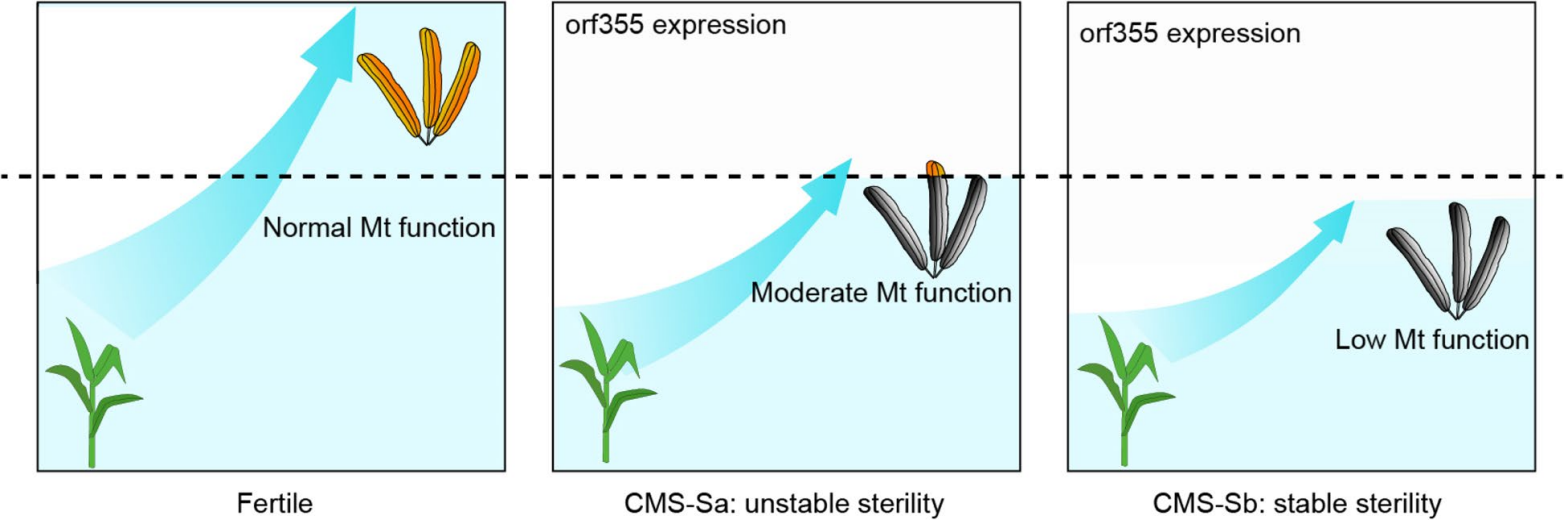
A hypothesis about the regulation of stability of sterility in CMS-S. Sterility in CMS-S is the consequence of inability of mitochondrial function to counteract the deleterious effect of *orf355*. Mitochondrial activity increases in anther tissue to meet the large amount energy required for pollen development, which is a representative example of mitochondrial robustness (indicated by blue arrows). However, mitochondrial activity varies among tissues and even among different cells of the same tissue. In anthers of CMS-Sa, microspores with moderate mitochondrial activity that higher enough to reach the threshold (dotted line) for normal pollen development can revert to being fertile. In CMS-Sb, the loss of one copy of *nad1*-exon1 reduces the mitochondrial robustness. The mitochondrial activity of all microspores could not reach the threshold required for normal pollen development. This defect may be sufficient to sustain the normal vegetative growth, but is manifested in specific situations such as sterility reversion

Although the precise molecular mechanism of CMS-S is as yet unknown, CMS-S maize is the only system where restorers are reported to arise in real time. This implies that the defect conferred by *orf355* may be not as severe as other reported CMS genes in stable CMS system [47–49]. *Orf355* expression increased during pollen development, leading to the gradual impairment of mitochondrial function [10]. The final degeneration of most microspores may result from the inability of mitochondria to counteract the deleterious effect of *orf355*. However, mitochondrial function varies among tissues and even among different cells in the same tissue. Thus, anthers in CMS-Sa with mitochondrial function that high enough to reach the threshold for normal pollen development can revert to being fertile. In this way, the unstable sterility in the CMS-Sa subtype probably reflects mitochondrial robustness that successfully counteract the deleterious effect of *orf355*. All reported maize mitochondrial genomes contain a basic suite of functional genes encoding components of macromolecular complexes, but the copy number of individual genes or exons varies among cytotypes [15, 23]. Variations in the copy number of these essential OXPHOS genes can affect transcription. We observed higher transcript levels of mitochondrial genes in the subtype with two copies of *nad1*-exon1, but lower levels in the subtype with one copy. We speculated that changes in OXPHOS gene transcription may affect translation or assembly efficiency of mitochondrial complex, ultimately affecting the functional robustness of the respiratory chain [50, 51]. The intact one copy of *nad1*-exon1 in CMS-Sb may be sufficient to meet the energy requirements for normal vegetative growth, but not to meet the increase energy requirements of pollen development. Thus, this defect is manifested only in specific situations such as sterility reversion where robust mitochondrial function is required (Fig. 8).

One point should be noted that the unstable sterility discussed here is different from the spontaneous reversion reported by previous studies. Usually, a fertile plant with CMS-S cytoplasm was caused by restorer-of-fertility gene such as *Rf3*, *Rf9* and *rfl1*, or by S1- and S2-plasmid integration into mitochondrial genome. Here, a slight modification of mitochondrial genome that is not associated with CMS gene exerts additive effect on male sterility expression when combined with the CMS gene. In other words, the deletion of *nad1*-exon1 in the second copy of R17 plays as an enhancer in terms of genetics. Practical observations indicated that most of these plants with exerted anthers did not fully revert to being fertile, although a small portion of the pollen in the exerted anthers was stainable with I_2_-KI solution. In addition, the exerted anthers in CMS-Sa usually appeared several days after natural pollination, so they would not meet the functional silks to complete fertilization. Nevertheless, the presence of starch granules in these pollen grains implied that they had the potential to germinate, and represented the partial recovery of mitochondrial function in the supposedly sterile pollen.

Another notable feature of the two newly sequenced mitochondrial genomes is the nucleotide substitution bias in plastid-derived sequences. One important feature of angiosperm mitochondrial genomes is that they have acquired foreign DNA from diverse sources, especially by intracellular gene transfer from the plastids [52–54]. Although most of the foreign plastid DNA results in non-functional sequences, some of them play important role during plant development, e.g., providing tRNAs in mitochondrial protein synthesis, the creation of promoter regions and codons, and the participation in post-transcriptional RNA processing [55–57]. In this study, we detected higher nucleotide mutation rates in *mtpt* sequences than in non-*mtpt* regions. In addition, we detected a 3.3-kb sequence deletion adjacent to a 411-bp *mtpt* sequence. One explanation for this result is that the *mtpt* region is recombinationally active and the transfer of plastid DNA into the mitochondrion is a relatively common event [23]. In this scenario, *mtpt* sequences would recombine with homologous DNA from the plastid. In rare cases, replication of *mtpt* sequences may fail to utilize the recombinational repair machinery, resulting in the occasional introduction of mutations into the mitochondrial genome.

In this study, we found that *mtpt* sequences may serve as recombinationally active sites in mediating mitochondrial genome rearrangement, leading to the copy number variation of mitochondrial gene. In addition, our comparative analyses provide evidence for a relationship between the copy number of mitochondrial gene and functional plasticity, which gives new insights into the stability of male sterility in maize CMS-S.

## Supplementary Information

**Additional file 1.**

**Additional file 2.**

**Additional file 3.**

**Additional file 4.**

**Additional file 5.**

**Additional file 6.**

**Additional file 7.**

**Additional file 8.**

**Additional file 9.**

**Additional file 10.**

**Additional file 11.**

**Additional file 12.**

## Data Availability

The obtained sequence data have been deposited into NCBI database with Accession No. PRJNA864113, https://www.ncbi.nlm.nih.gov/sra/PRJNA864113.
