# Supplementary material for: Comparative analysis of mitochondrial genomes of maize CMS-S subtypes provides new insights into male sterility stability
Source: BMC Plant Biol. 2022 Oct 1;22:469. doi: 10.1186/s12870-022-03849-6 (PMC9526321; doi:10.1186/s12870-022-03849-6)
Supplement: Supplementary file 1 — Additional file 1. [file 12870_2022_3849_MOESM1_ESM.pdf]

Supplemental Table S1. The primer sequences used in this study.

The primer sequences used for qRT-PCR assays in mitochondrial gene expression

	Forward sequence (5'-3')	Reverse sequence (5'-3’)
cox1	aacgacgaatcccaactacg	ttcccatgcatttcttaggg
cox2	ctggatcatcttctggaatcg	ttcccatgcatttcttaggg
cox3	gcaactggctttcatggttt	cgtgatgcttcttggtcaga
cob	gttgacatccgatccaacct	ccgcaatagcaccagttttt
rps1	ccgcttcttcttccacaaag	atgaaacctgcgatggctac
rps2A	ttgggtcatccccagagtag	aaggctctcattgacgcact
rps2B	cagaacacctcgttcccatt	gttggccactacagggagaa
rps3	gagacgaaagccaaaggtga	ttcagatccaagtcggttca
rps4	aggggatgccgcttactact	cagcgaaggaactgcctaag
rps7	tacatcgcgttcagttcgag	tcgcatgatcgatggtaaaa
rps13	gagttcgttgaccgcgtaat	gggaattgaagaggggagaa
rpl16	ttcgcgacccctactacatc	tgacggaatttcaggtgtca
ccmB	gtcgtcacgcccttaatgat	gaaaaggttcgggaggaaag
ccmC	aagggagattcgggaaaaga	atgttcctatgcccattcca
ccmFC	cacatggaggagtgtgcatc	caaaggcaatcgttgagtga
ccmFN	catctggcacgagatgaaaa	tcgaacaaaaggatcggaac
matr	ctttcgttttccctccttcc	taccattgaattcggccttc
mttB	ggggaggagttaggaacagg	ctatggggaacaaaggcaga
atp1	cccggggatgttttctattt	cgtctccagcttgtgtttca
atp4	tttgtgcatcaagtccgaag	cccgtcgagagtttctttga
atp6	tccggaaatgtgaaacacaa	tcataccctggggattacga
atp8	ccgtcgactatttgggaaaa	caacaagtgatccgactgga
atp9	gcgaaatccttcattggcta	atcagaaaggccatcattgg
orf77	ttcgttttccgatgcataaa	cagtgaaagaagcccgctaa
orf355	ttacgaggctgatgcaagtg	tgtggcctcgattagtaccc
orf134b	tcaaggacatccggataagg	tcaagccatcgactgaacac
orf147b	agcctccccaatggttatgt	atgaaaggctggagcaacac
orf189	ccgatgaaagggtcgtagaa	gtattcgttcggctgcaact
orf191	cccaggggtctcttcctatc	atgaaaacgggcatatcgaa
orf424	cgagccctccccttatttag	acggaaagcgcttatttcct
nad1	aatacggggaacaagggaat	tctgcagctcaaatggtctc
nad2	gccaagattttgaccggata	ggtgggtgaaccctcataga
nad4	agggctgaagaaaccagtcc	agcccatatgaatttggtga
nad4L	ttcggggaatcctccttaat	tcatccaaagaaacggaaaa
nad5	cgtacacattccgacgattg	cccacatacgagaaaaggtca
nad6	tccgtctggtttttggtttc	ggcatttcgtcggaatacat
nad7	aagaggtcctggggtatgct	tgtcgcatctcttcgatacg
nad9	caagtgcggacgaagtaaca	aaatccggatgattgatgga
18S	cagctcgtgtcgtgagatgt	ttctccttaggcgcatgtct

The primer sequences used in *mtpt* and platid sequence amplification

	Forward sequence (5'-3')	Reverse sequence (5'-3’)
Mt-Fragment 1	agacccggatacctccctta	aggggaacggaaccgtatag
Mt-Fragment 2	aagaagagccgaagccctac	gtcagggatttctccccatt
Mt-Fragment 3-1	ttgctattggacggctttct	cggttctgtggagtggtttt
Mt-Fragment 3-2	ctttggattgtcttcaattgga	tagatctgcgcctgccttta
Mt-Fragment 3-3	ttttcctccacgtcgatagg	tccacatgtcgagattgcat
Mt-Fragment 4	ctatttatccggccagtgga	tgttacggcctaaatgctga
Mt-Fragment 5	tgcatttttgcaattcattgat	cgcaattcaaaaacggaaat
Mt-Fragment 6	gattgtggtatccccacctc	tcacaaacggatccgaactt
Mt-Fragment 7	atcaccatcactccctctgc	ctactggcaccggcactatt
Mt-Fragment 8	cctaaaggaaagccggaaat	agagcagtcgattcggtcat

Mt-Fragment 9-1	atcaccttacgcagccaaac	aggccctgaaaggagaagag
Mt-Fragment 9-2	cgatttgtcaaagggattgg	atggagtcgagatccattgc
Mt-Fragment 10-1	cgtattgaatgcgcaaagtg	aatgggaaatgccctacctt
Mt-Fragment 10-2	cacttgctgccgttactcaa	aaggggaacgcgaaataact
Mt-Fragment 11	ccaaagatttcggtcagagc	tcttcattaggcaggcaggt
Pt-Fragment 1	ttcatcgaatacggctttcc	ggagcaggctaccatgagac
Pt-Fragment 2	tgtgcttgtggaaactctcg	aaatgtttcgccaattgcat
Pt-Fragment 3-1	cgcaaaatggagaaaatcgt	gcacttgaaatagccgaagc
Pt-Fragment 3-2	tggattgtcttcatcaattgga	cctatcgacgtggaggaaaa
Pt-Fragment 3-3	ccaaacagtccgatttttcc	caaatacgttacccacaatgga
Pt-Fragment 4	gaagggcaatcactcgttct	cccctgttatttttgggtca
Pt-Fragment 5	ggcaagctcgtacacaatga	attgatcacaagccgaaaca
Pt-Fragment 6	cacaaaggagtgcgacaaga	aagggcacctaatctcacctc
Pt-Fragment 7	ccactctggaagagctcagg	taaaggtccttcccccctttg
Pt-Fragment 8	tccaaagcatacggctttct	ggttcaattcctgctggatg
Pt-Fragment 9-1	tgaaaaagtgggtcgcttct	tcggatcgggtgaattagag
Pt-Fragment 9-2	aggccctgaaaggagaagag	tcaatgaaccccattcttgc
Pt-Fragment 10	tcggttggaacttttcttgc	ataagccagatgacggaacg
Pt-Fragment 11	cctggggacccagatcaata	gaatacccagttgaagggtaatg

The primer sequences used for determination of mitochondrial DNA level

	Forward sequence (5'-3')	Reverse sequence (5'-3’)
C33-C34	ttatcgaatccatgggggacttgtca	gacctatcgcgtggtaaagag
C35-C36	caactaaatgaaataataggaggggat	gggccattcttatgtcatgggct
C39-C40	cagcactgagttgagtagcgac	catccccacatgcgattgc

The primer sequences used for Circular RT-PCR assays

	Sequence (5'-3')
nad1-reverse transcript primer	gcccaagcgaccagac
nad1-exon1-F1	caagcgagcgtacctttgtt
nad1-exon1-F2	ggaaacagcaactaactatgg
nad1-exon1-R1	accggtggagaggctattct
nad1-exon1-R2	tttcgcctgccttcc
nad2-reverse transcript primer	tccagcattacggcaa
nad2-exon4-5-F1	tgaaaacgctcctaacccct
nad2-exon4-5-F2	actactagcaatgacttcctctt
nad2-exon4-5-R1	ttactttcgtttgggccatgg
nad2-exon4-5-R2	ggtgggtgaaccctcataga
nad2-exon3-4-5-F1	tgaaaacgctcctaacccct
nad2-exon3-4-5-F2	actactagcaatgacttcctctt
nad2-exon3-4-5-R1	accacccatcctaccctact
nad2-exon3-4-5-R2	gtggtggttgggcctac
