# Supplementary material for: Comparative analysis of mitochondrial genomes of maize CMS-S subtypes provides new insights into male sterility stability
Source: BMC Plant Biol. 2022 Oct 1;22:469. doi: 10.1186/s12870-022-03849-6 (PMC9526321; doi:10.1186/s12870-022-03849-6)
Supplement: Supplementary file 2 — Additional file 2. [file 12870_2022_3849_MOESM2_ESM.pdf]

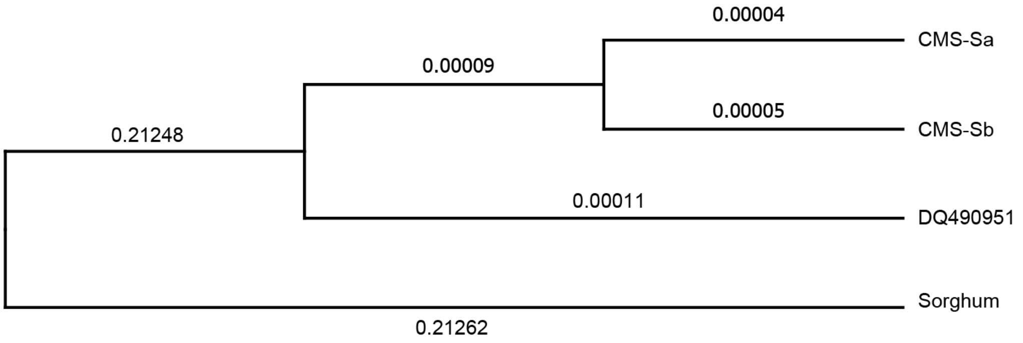

Figure S1. A phylogenetic tree relating the mitochondrial genomes of maize CMS-S subtypes. The mitochondrial genomes of CMS-Sa, CMS-Sb and DQ490951 were used to build an evolutionary tree, mitochondrial genome of Sorghum (DQ984518) was used as the outgroup. The labeled number indicated distance among each genome. The tree was drawn using R script.
