# Supplementary material for: Comparative analysis of mitochondrial genomes of maize CMS-S subtypes provides new insights into male sterility stability
Source: BMC Plant Biol. 2022 Oct 1;22:469. doi: 10.1186/s12870-022-03849-6 (PMC9526321; doi:10.1186/s12870-022-03849-6)
Supplement: Supplementary file 3 — Additional file 3. [file 12870_2022_3849_MOESM3_ESM.pdf]

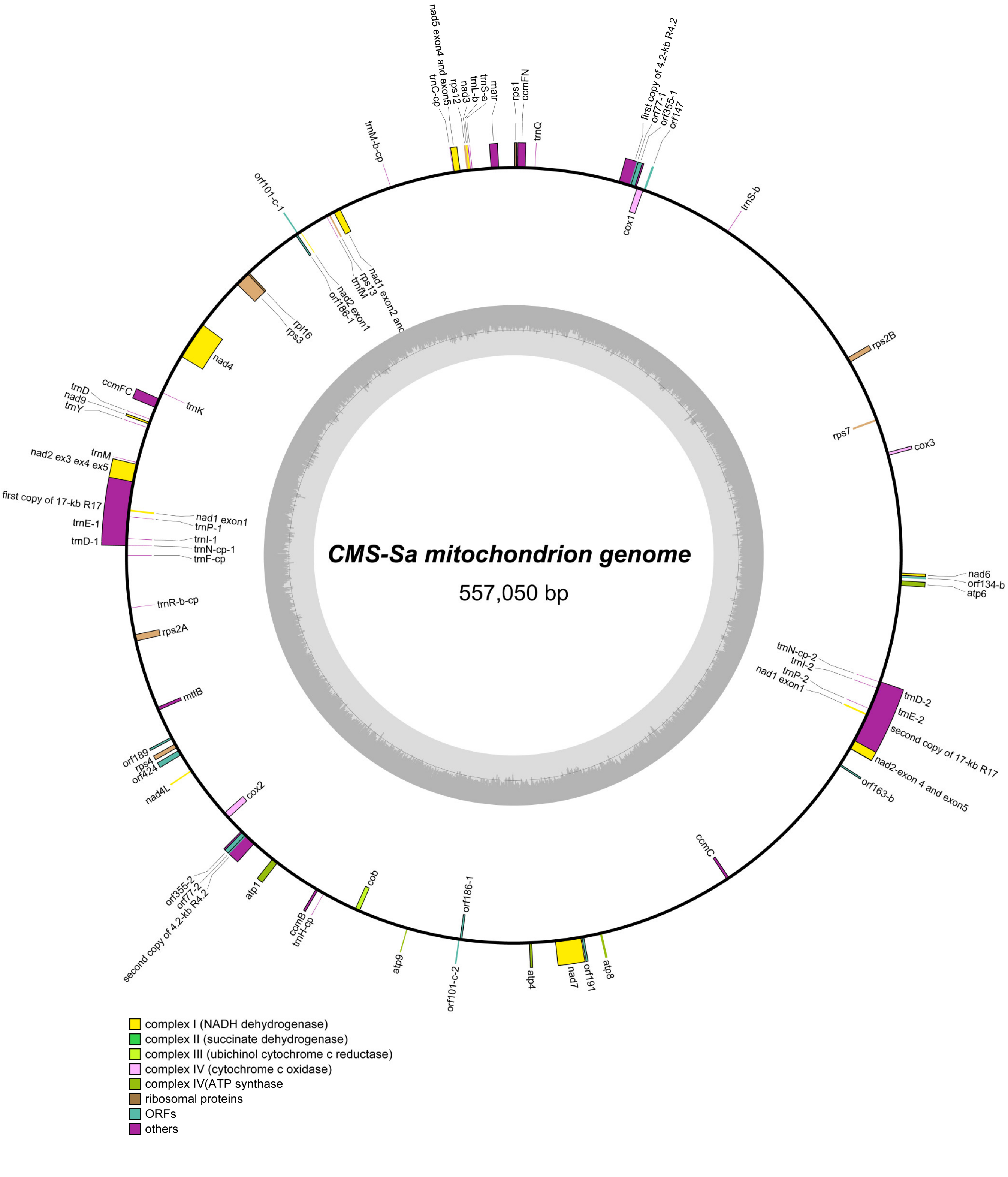

Figure S2. Graphical mitochondrial genome maps of subtype CMS-Sa. All of the known genes were annotated with different colors according their function.
