# Supplementary figures and images for: Comparative analysis of mitochondrial genomes of maize CMS-S subtypes provides new insights into male sterility stability
Source: BMC Plant Biol. 2022 Oct 1;22:469. doi: 10.1186/s12870-022-03849-6 (PMC9526321; doi:10.1186/s12870-022-03849-6)

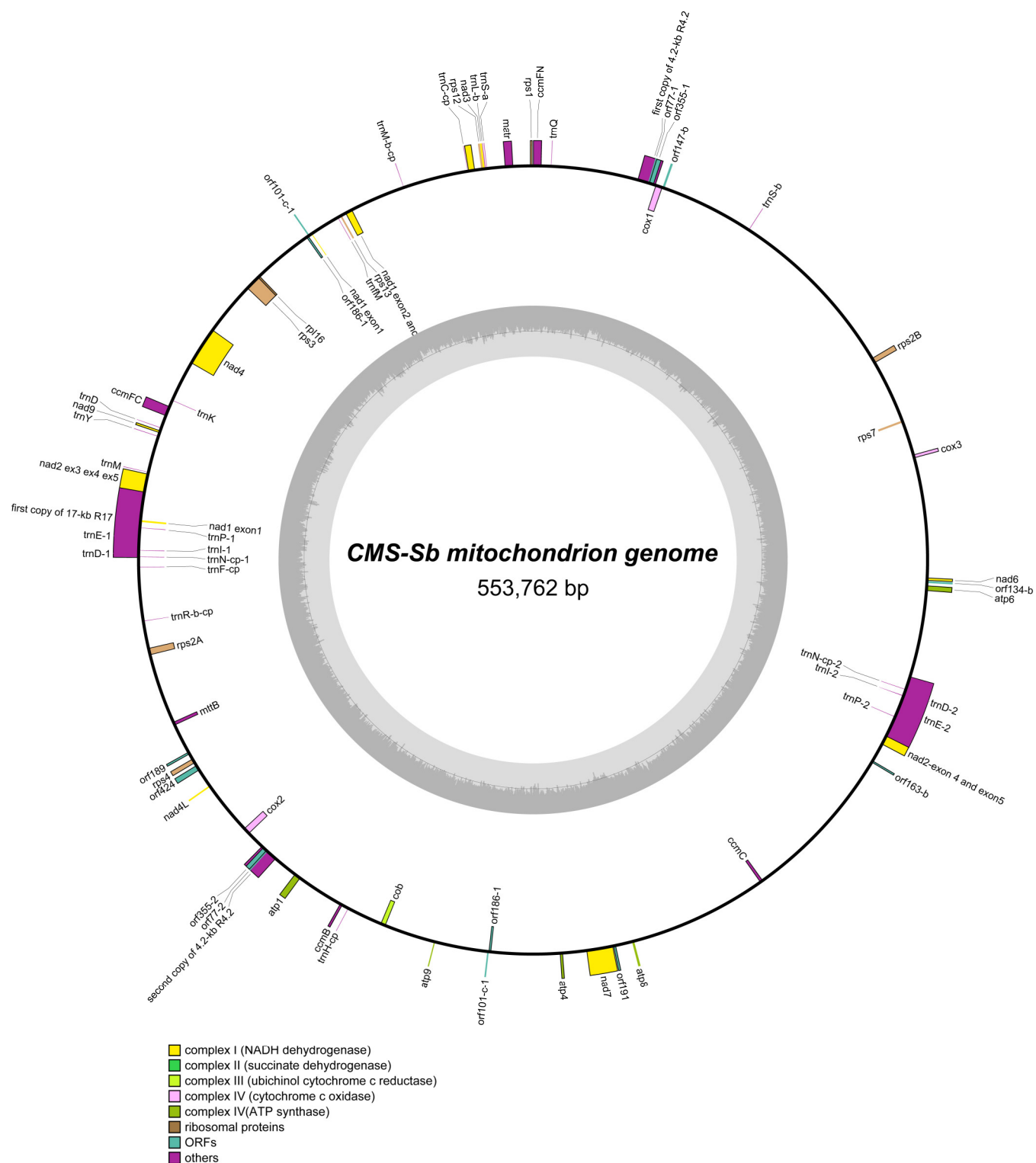

Supplement: Supplementary file 4 — Additional file 4. [file 12870_2022_3849_MOESM4_ESM.pdf]

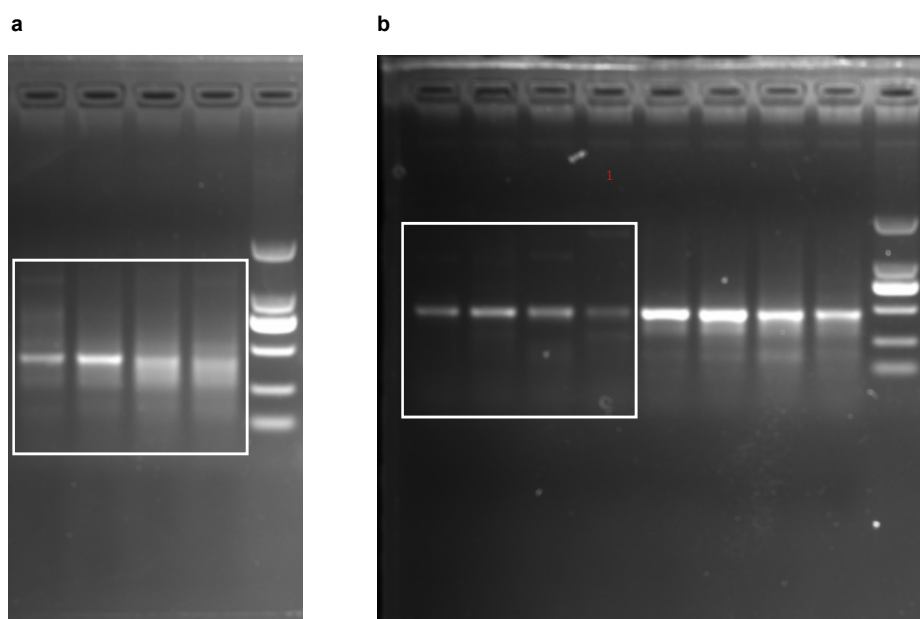

**Figure S4.** Uncropped gel of Fig.6b (a) and 6c (b). The cropped areas were labeled with white rectangles.

Supplement: Supplementary file 8 — Additional file 8. [file 12870_2022_3849_MOESM8_ESM.pdf]
