# Supplementary material for: Comparative analysis of mitochondrial genomes of maize CMS-S subtypes provides new insights into male sterility stability
Source: BMC Plant Biol. 2022 Oct 1;22:469. doi: 10.1186/s12870-022-03849-6 (PMC9526321; doi:10.1186/s12870-022-03849-6)
Supplement: Supplementary file 5 — Additional file 5. [file 12870_2022_3849_MOESM5_ESM.pdf]

Supplemental Table S2. Nucleotide substitution in CMS-Sa and CMS-Sb, compared with the reference genome.

Mitochondrial genome								Plastid genome		
PCR Fragment	Coordinates	Reference	CMS-Sa	CMS-Sb	*mtpt*	Size (bp)	Feture/Gene	Size (bp)	Feature/Gene	PCR Fragment
	1040	A	T	A	-					
	1938	GGG	AGA	AGA	-					
Mt-Fragment 1 4016 - 3847	3863	C	A	A	+	170		168		Pt-Fragment 1 92546 - 92713
	3890	C	A	A						
	3894	T	A	A						
	3897	G	A	A						
	3911	C	A	A						
	3918	C	A	A						
	7645	A	C	A	-					
	90977	CT	AA	AA	-					
Mt-Fragment 2 101352 - 100875	100924	C	A	A	+	478		444	*rpoC1 exon*	Pt-Fragment 2 24989 - 25432
	100929	CG	AA	AA						
	100962	C	A	A						
	100968	C	A	A						
	101030	C	A	A						
	101082	G	A	A						
	101099	C	A	A						
	101175	G	A	A						
	101178	G	A	A						
	101213	T	A	A						
	101282	C	A	A						
	101333	G	A	A						
	104694	A	A	T	-					
Mt-Fragment 3 166797 - 170522	166897	T	A	A	+	3726	*trnM-b-cp,next to 5.3kb repeat*	3681	*atp8 atpE trnM trnV*	Pt-Fragment 3 52990 - 56670
	166916	T	A	A						
	167018	G	A	A						
	167975	T	C	C						
	168386	A	C	C						
	168395	A	C	C						
	168752	A	T	T						
	168809	T	G	G						
	168823	T	C	C						
	169152	TCTGC	ATAGTT	ATAGTT						
	169244	G	T	T						
	169931	AAA	TTC	TTC						
	170013	A	C	C						
	170131	C	A	A						
	170308	C	T	T						
	203604	A	T	A	-					
Mt-Fragment 4 221967 - 220717	221888	G	A	A	+	1251		1211	*ZemaCp157*	Pt-Fragment 4 98699 - 99909
	240296	A	C	A	-					
	248625	A	C	C	-					
	251319	C	A	A	-					
	261847	A	T	A	-					
	264739	A	C	A	-					
Mt-Fragment 5 266110 - 265700	265632	C	A	C	+	411	*nad1-intron*	425	*ndhE*	Pt-Fragment 5 112176 - 112600
	265739	G	A	G						
	265856	T	A	T						
	265871	T	A	T						
	266013	G	A	G						
Mt-Fragment 6 278415 - 278623	278420	G	A	A	+	209	*trnF-cp*	215	*trnF*	Pt-Fragment 6 49796 - 50010
	278423	T	A	A						
	278437	GAGT	AAAA	AAAA						
	278455	C	A	A						
	278462	G	A	A						
	278571	C	A	A						
	278577	G	A	A						
	278590	T	A	A						
	278611	G	A	A						
	283083	CT	AA	AA	-					
Mt-Fragment 7 292113 - 290658	290903	C	A	A	+	1456	*trnR-b-cp*	1536	*ZemaCr114 ZemaCr115 ZemaCr116 trnR*	Pt-Fragment 7 101365 - 102900
	291493	C	A	A						
	291808	G	T	T						
	291931	C	A	A						
	305449	C	A	C	-					
	334046	A	T	A	-					
Mt-Fragment 8 340230 - 340089	340015	C	A	A	+	142		150		Pt-Fragment 8 84716 - 84865
	340033	G	A	A						
	340044	G	A	A						
Mt-Fragment 9 364122 - 360938	361015	G	C	C	+	3185	*trnL-c-cp*	3189	*ZemaCp071 ZemaCp072 ZemaCp073 ZemaCp074 trnL*	Pt-Fragment 9 86083 - 89271
	361248	A	G	G						
	361271	TTTT	G	G						
	361289	T	G	G						
	362776	T	G	G						
	363457	T	G	G						
	363628	T	G	G						

Mt-Fragment 10<br>372096 - 374315	370923	A	C	A	+	2220	*trnH-cp*	2235	*rpl23*<br>*rpl2*<br>*ZemaCp064*<br>*rps19*<br>*ZemaCp066*<br>*trnH*	Pt-Fragment 10<br>82476 - 84710
	372004	C	A	A						
	372008	C	A	A						
	372033	C	A	A						
	372145	T	A	A						
	372308	C	A	A						
	372674	T	G	G						
	373234	G	A	A						
	373252	A	C	C						
	373257	G	T	T						
	373354	A	C	C						
	373357	G	T	T						
	373619	G	T	T						
	373691	C	T	T						
	373734	A	C	C						
	373939	G	T	T						
Mt-Fragment 11<br>376209 - 374030	374115	G	T	T	+	2040		2152	*rbcL*	Pt-Fragment 11<br>56669 - 58820
	374241	T	A	G						
	374270	A	A	C						
	374277	A	A	C						
	374456	C	A	T						
	374501	G	A	T						
	374800	A	T	T						
	374821	T	G	G						
	375147	A	C	C						
	375196	G	A	A						
	375977	G	A	A						
	381219	A	C	A	-					
	406458	A	T	A	-					
	420703	C	A	A	-					
	440951	G	T	G	-					
	468974	A	C	A	-					
	491209	G	A	A	-					
	509601	G	A	G	-					
	509628	C	A	A	-					
	509642	C	A	A	-					
	509645	T	A	A	-					
	509651	T	A	A	-					
	509668	G	A	A	-					
	509683	GCC	AAA	AAA	-					
	510754	C	A	A	-					
	513184	A	T	A	-					
	516074	A	C	A	-					
Mt-Fragment 12<br>517469 - 517059	517076	G	G	T	+	411		425	*ndhE*	Pt-Fragment 12<br>112176 - 112600
	517150	AA	AA	CT						
	517193	T	T	G						
	517208	T	T	G						
	517289	A	A	C						
	517350	G	G	T						
