# Supplementary material for: Comparative analysis of mitochondrial genomes of maize CMS-S subtypes provides new insights into male sterility stability
Source: BMC Plant Biol. 2022 Oct 1;22:469. doi: 10.1186/s12870-022-03849-6 (PMC9526321; doi:10.1186/s12870-022-03849-6)
Supplement: Supplementary file 6 — Additional file 6. [file 12870_2022_3849_MOESM6_ESM.pdf]

Supplemental Table S3. Indels within CMS-Sa and CMS-Sb, compared with the reference genome.

Genome location	Reference	CMS-Sa	CMS-Sb
167742	CATAA	-	-
168026	TGGAT	-	-
168228	TCTTG	-	-
168402	TTTAG	-	-
168422	-	AATTT	AATTT
169667	AACCC	-	-
170088	GAATC	-	-
170177	TGTGC	-	-
221289	TACGGGTACGG	-	-
239141	AAGAT	-	-
361708	AACC	-	-
373592	-	TTATAGC	TTATAGC
373631	CCAGAA	-	-
373699	TCTAC	-	-
373753	G	TTATGA	TTATGA
