# Supplementary material for: Comparative analysis of mitochondrial genomes of maize CMS-S subtypes provides new insights into male sterility stability
Source: BMC Plant Biol. 2022 Oct 1;22:469. doi: 10.1186/s12870-022-03849-6 (PMC9526321; doi:10.1186/s12870-022-03849-6)
Supplement: Supplementary file 7 — Additional file 7. [file 12870_2022_3849_MOESM7_ESM.pdf]

Supplemental Table S4. Simple sequence repeats variation in CMS-Sa and CMS-Sb.

Genome location	Repeat unit	Repeat number		
		Reference	CMS-Sa	CMS-Sb
129949	AAAGT	7	5	6
133509	AAAGT	7	7	5
240619	ATAAGA	10	9	8
245228	ATAGAA	6	4	6
291709	TACAG	2	1	1
362507	GCCT	2	1	1
372457	CTTAC	2	1	1
383334	GGCCA	3	2	3
394795	TAGGGT	9	9	8
